# Modelling Blood Flow and Metabolism in the Piglet Brain During Hypoxia-Ischaemia: Simulating Brain Energetics

**DOI:** 10.1007/978-1-4614-7411-1_45

**Published:** 2013-03-25

**Authors:** Tracy Moroz, Tharindi Hapuarachchi, Alan Bainbridge, David Price, Ernest Cady, Ether Baer, Kevin Broad, Mojgan Ezzati, David Thomas, Xavier Golay, Nicola J. Robertson, Chris E. Cooper, Ilias Tachtsidis

**Affiliations:** 00451 CoMPLEX, University College London, London, UK; 00452 Medical Physics and Bioengineering, University College London Hospitals, London, UK; 00453 Department of Medical Physics and Bioengineering, University College London, London, UK; 00454 Institute for Women’s Health, University College London, London, UK; 00455 Institute of Neurology, University College London, London, UK; 00456 School of Biological Sciences, University of Essex, Colchester, UK

**Keywords:** Cerebral Blood Flow, Magnetic Resonance Spectroscopy, Measured Signal, Mean Arterial Blood Pressure, Oxygen Extraction Fraction

## Abstract

We have developed a computational model to simulate hypoxia-ischaemia (HI) in the neonatal piglet brain. It has been extended from a previous model by adding the simulation of carotid artery occlusion and including pH changes in the cytoplasm. Here, simulations from the model are compared with near-infrared spectroscopy (NIRS) and phosphorus magnetic resonance spectroscopy (MRS) measurements from two piglets during HI and short-term recovery. One of these piglets showed incomplete recovery after HI, and this is modelled by considering some of the cells to be dead. This is consistent with the results from MRS and the redox state of cytochrome-c-oxidase as measured by NIRS. However, the simulations do not match the NIRS haemoglobin measurements. The model therefore predicts that further physiological changes must also be taking place if the hypothesis of dead cells is correct.

## Introduction

Hypoxia-ischaemia (HI) is a major cause of brain damage in neonates. Piglets are often used as models to investigate the processes occurring during HI and to test treatments. We have previously developed a computational model to simulate oxygen deprivation in the neonatal piglet brain [1]. This model has been extended to allow simulations of HI induced by carotid artery occlusion. We are able to use the model to compare with data from near-infrared spectroscopy (NIRS) and magnetic resonance spectroscopy (MRS). These two non-invasive modalities have been used simultaneously to monitor newborn piglets subjected to HI. The model allows the measurements to be analysed together and the relationships between them to be explored.

## The Model

**Fig. 45.1:**
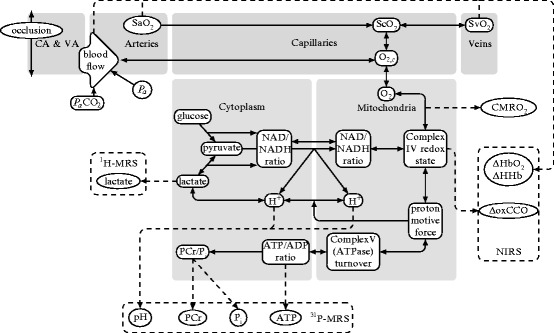
Schematic diagram of the model. CA and VA refer to the carotid and vertebral arteries

The model simulates circulation and metabolism in the neonatal brain. It is an extension of a model which has previously been used to investigate anoxia in piglets [1]. A schematic diagram of the model is shown in Fig. 45.1. The metabolic part of the model simulates metabolites both in the cytoplasm and the mitochondria. The mitochondrial part of the model focuses on the redox state of the electron transport chain, in particular cytochrome-c-oxidase (CCO). The cytoplasmic part of the model focuses on energy metabolism and includes simplified descriptions of glycolysis and ATP use. The model is able to simulate the variables which are measured by MRS including ATP, phosphocreatine (PCr), inorganic phosphate (P_i_) and lactate concentrations. It has also been extended to simulate pH changes in the cytoplasm.

The circulatory part of the model allows simulation of the NIRS haemogolobin measurements. It has been extended to allow simulation of carotid artery occlusion. This was done by adding an extra compartment to represent all the arteries supplying the brain. The main arteries responsible for this are the carotid arteries: in adult humans it is estimated that 80 % of the cerebral blood supply flows through them [2]. In the model, this fraction (*c*_*f*_) determines the conductance of the supplying arterial compartment (*G*_0_) during carotid artery occlusion by

45.1 $$ {G}_{0}={G}_{0,\text{n}}(1-{c}_{f})$$

where *G*_0,*n*_ is the conductance when there is no occlusion which is set by

**Fig. 45.2:**
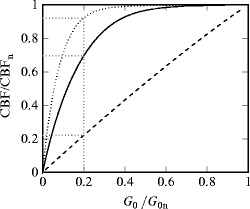
CBF versus conductance of the supplying arterial compartment for *G*_*f*_ = 10.0 (*dotted*), *G*_*f*_ = 4.0 (*solid*) and *G*_*f*_ = 0.1 (*dashed*). Both variables are shown as fractions of their normal value. Complete occlusion of the carotid arteries is equivalent to reducing the conductance to 0.2

45.2 $$ {G}_{0}={G}_{f}{G}_{n}$$

where *G*_*n*_ is the normal conductance of the cerebral arterial compartment. The ratio *G*_*f*_ is difficult to obtain from the literature, so was set by examining the results of the simulations. The change in modelled cerebral blood flow (CBF) as a function of *G*_0_/*G*_0,*n*_ is shown in Fig. 45.2. Three different values for the fraction *G*_*f*_ are shown. When the *G*_*f*_ is large, the CBF remains high until the conductance is only a small fraction of its normal value. When *G*_*f*_ is small, the relationship becomes more linear. From examining this curve, a value of 4 was chosen for *G*_0_.

## Methods

The simulations were compared with modelled data from experiments involving piglets less than 24-h-old. The piglets were anaesthetised and mechanically ventilated. Their arterial oxygen saturation (SaO_2_) and mean arterial blood pressure (MABP) were continuously monitored. The piglets were also monitored with NIRS to measure the change in concentration of oxyhaemoglobin (ΔHbO_2_), deoxyhaemoglobin (ΔHHb) and oxidised cytochrome-c-oxidase (ΔoxCCO). In addition, measurements of nucleotide triphosphate (NTP) which is mainly ATP, PCr and P_i_ were recorded as a fraction of the exchangeable phosphate pool (EPP) by ^31^P-MRS. After 10 min of baseline measurements, vascular occluders surrounding both carotid arteries were inflated and the inspired oxygen fraction (FiO_2_) was reduced to 12 %. When the β-NTP peak had fallen to 50 % of its baseline value, FiO_2_ was titrated to maintain the β-NTP peak between 30 % and 50 % of its baseline height for 12.5 min. Following this, the occluders were deflated and FiO_2_ was returned to normal. Measurements were continued for approximately another 2 h.

The measured SaO_2_ and MABP were used as inputs to the model, and its outputs were compared with the NIRS and MRS measured variables. A Morris sensitivity analysis was used to identify which parameters had the most important effect on fitting the modelled signals to the measured signals. The results showed that the most important parameters were those representing the concentration of the measured quantities, i.e., the blood haemoglobin concentration, the tissue concentration of cytochrome-c-oxidase and the normal concentrations of ATP, PCr and P_i_. These parameters were adjusted to best match the modelled and measured signals for the individual piglets.

Not all piglets showed recovery of the ΔoxCCO signal and the ^31^P-MRS signals following the insult. One hypothesis to explain this is that some of the cells have died. In order to simulate this, the model was altered so that a fraction of the cells *d* were treated as dead following the insult. In these cells, CCO was assumed to be completely reduced and all exchangeable phosphate was assumed to be in the form of P_i_. It was also assumed that no oxygen was consumed in the dead cells, so that the modelled rate of oxygen transfer from the capillaries to the mitochondria was reduced to 1 − *d* of its normal rate. Several of the model outputs were also changed:

45.3 $$ \begin{array}{l}\rm{output}\rm{}\rm{NTP}/\rm{EPP}=\frac{(1-d)[\rm{ATP}]}{[\rm{EPP}]}\\
\rm{output}\rm{}\rm{PCr}/\rm{EPP}=\frac{(1-d)[\rm{PCr}]}{[\rm{EPP}]}\\
\rm{output}\rm{}{\rm{P}}_{\rm{i}}/\rm{EPP}=\frac{(1-d)[\rm{Pi}]}{[\rm{EPP}]}+d\\
\rm{output}\rm{}\Delta \rm{oxCCO}=(1-d)\Delta \rm{oxCCO}-d\rm{oxCCO}\\
\rm{output}\rm{}{\rm{CMRO}}_{2}=(1-d){\rm{CMRO}}_{2}.\end{array}$$

## Results

**Fig. 45.3:**
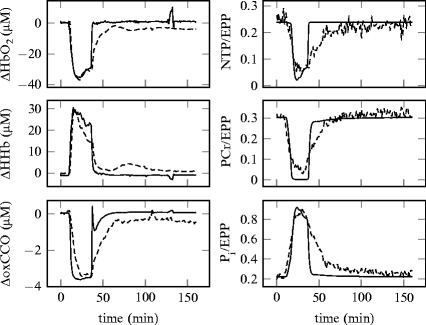
A comparison between modelled (*solid*) and measured (*dashed*) signals from NIRS (*left*) and MRS (*right*) from a single piglet (LWP180)

**Fig. 45.4:**
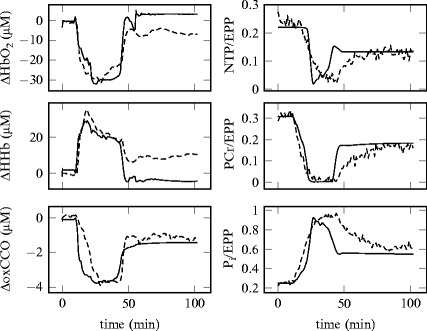
Modelled signals (*solid*) compared with measured signals (*dashed*) from NIRS (*left*) and MRS (*right*) from a piglet (LWP188) which did not recover following HI. The simulations use a value of *d* = 0.4 after the insult

Figure 45.3 shows the simulated and measured signals for a piglet (LWP180) which showed recovery following HI. The fraction of dead cells *d* was set to 0. Figure 45.4 shows the same signals but for a piglet (LWP188) which did not recover. For these simulations, *d* was set to 0.4.

## Discussion

The model has been used to simulate NIRS and MRS measurements during HI. The model is able to simulate carotid artery occlusion. It is known that with only one carotid artery occluded, there is no change in CBF in piglets. Measurements of CBF when both arteries are occluded (and there is no change in oxygen saturation) include 75 % [3] and 45 % [4] of the baseline value. However, these experiments also involved changes in blood pressure. The modelled value lies between these two values, but more data are necessary to validate this part of the model.

The model is well able to simulate the magnitude of changes during HI. The time course of all the metabolic signals show that the model is predicting the recovery of these signals to baseline faster than is seen in the measured signals. A possible reason for this is that there are physiological changes occurring during HI which are not modelled.

The difference in recovery time is even more pronounced in the piglet which did not fully recover. However, the final values of the modelled ΔoxCCO, NTP/EPP, PCr/EPP and P_i_/EPP are similar to their measured equivalents. This is consistent with a fraction of the cells being dead. The model allows the consequences of this assumption on other signals to be investigated. It predicts that the overall rate of oxygen metabolism (CMRO_2_) would drop compared to baseline, which would cause the oxygen extraction fraction to fall and hence ΔHbO_2_ to rise and ΔHHb to fall as seen in Fig. 45.4. However, this is not what is seen in the measurements, which suggests that there are other physiological changes occurring after HI if the assumption of cell death is correct. Possibilities for this include a large increase in CMRO_2_ in the functioning cells, perhaps caused by mitochondrial uncoupling, or that blood may no longer be perfusing the whole brain. Alternatively, the cells may not be dead but functioning at a reduced capacity, or spatial differences between the measurements and pattern of cell death may give misleading results. Finally, the experimental results may have been affected by changes in the haematocrit of the piglet. Further investigation with the model and analysis of data from more piglets will help to answer these questions.

## References

[1] Moroz T, Banaji M, Robertson NJ, Cooper CE, Tachtsidis I (2012). Computational modelling of the piglet brain to
simulate near-infrared spectroscopy and magnetic resonance spectroscopy data
collected during oxygen deprivation. J R Soc Interface.

[2] Edvinsson L, Mackenzie E, Mcculloch J (1992). Cerebral blood flow and metabolism.

[3] Kurth C, Levy W, McCann J (2002). Near-infrared spectroscopy cerebral oxygen saturation
thresholds for hypoxia-ischemia in piglets. J Cereb Blood Flow Metab.

[4] Oriot D, Beharry K, Gordon JB, Aranda JV (1995). Ascorbic acid during cerebral ischemia in newborn
piglets. Acta Paediatr.

